# How to treat mixed behavior segments in supervised machine learning of behavioural modes from inertial measurement data

**DOI:** 10.1186/s40462-024-00485-7

**Published:** 2024-06-10

**Authors:** Yehezkel S. Resheff, Hanna M. Bensch, Markus Zöttl, Roi Harel, Akiko Matsumoto-Oda, Margaret C. Crofoot, Sara Gomez, Luca Börger, Shay Rotics

**Affiliations:** 1 Hebrew University Business School, The Hebrew University of Jerusalem, Jerusalem, Israel; 2 Department of Biology and Environmental Science, Centre for Ecology and Evolution in Microbial Model Systems (EEMIS), Linnaeus University, 391 82 Kalmar, Sweden; 3 Kalahari Research Centre, Kuruman River Reserve, Van Zylsrus, South Africa; 4 Department for the Ecology of Animal Societies, Max Planck Institute of Animal Behavior, Constance, Germany; 5 Department of Biology, University of Konstanz, Constance, Germany; 6 Centre for the Advanced Study of Collective Behaviour, University of Konstanz, Constance, Germany; 7 Mpala Research Centre, Nanyuki, Kenya; 8 Graduate School of Tourism Sciences, University of the Ryukyus, Nakagami, Okinawa Japan; 9 Department of Biosciences, Swansea University, Swansea, Wales UK; 10 School of Zoology, Faculty of Life Sciences, and the Steinhardt Museum of Natural History, Tel Aviv University, Tel Aviv, Israel; 11 Kuruman River Reserve, Kalahari Research Centre, Van Zylsrus, South Africa

**Keywords:** Body-acceleration, Bio-logging, Machine learning, Animal behaviour

## Abstract

The application of supervised machine learning methods to identify behavioural modes from inertial measurements of bio-loggers has become a standard tool in behavioural ecology. Several design choices can affect the accuracy of identifying the behavioural modes. One such choice is the inclusion or exclusion of segments consisting of more than a single behaviour (mixed segments) in the machine learning model training data. Currently, the common practice is to ignore such segments during model training. In this paper we tested the hypothesis that including mixed segments in model training will improve accuracy, as the model would perform better in identifying them in the test data. We test this hypothesis using a series of data simulations on four datasets of accelerometer data coupled with behaviour observations, obtained from four study species (Damaraland mole-rats, meerkats, olive baboons, polar bears). Results show that when a substantial proportion of the test data are mixed behaviour segments (above ~ 10%), including mixed segments in machine learning model training improves the accuracy of classification. These results were consistent across the four study species, and robust to changes in segment length, sample size, and degree of mixture within the mixed segments. However, we also find that in some cases (particularly in baboons) models trained with mixed segments show reduced accuracy in classifying test data containing only single behaviour (pure) segments, compared to models trained without mixed segments. Based on these results, we recommend that when the classification model is expected to deal with a substantial proportion of mixed behaviour segments (> 10%), it is beneficial to include them in model training, otherwise, it is unnecessary but also not harmful. The exception is when there is a basis to assume that the training data contains a higher rate of mixed segments than the actual (unobserved) data to be classified—such a situation may occur particularly when training data are collected in captivity and used to classify data from the wild. In this case, excess inclusion of mixed segments in training data should probably be avoided.

**Supplementary Information:**

The online version contains supplementary material available at 10.1186/s40462-024-00485-7.

## Introduction

Our ability to study wild animal behaviour has been revolutionized over the past decade by the utilization of animal-attached bio-logging devices [1, 2]. Among the primary data types recorded by such devices are location (GPS) and inertial measurements (particularly body-acceleration) from which behavioural modes can be inferred [3, 4]. This inference is usually carried out using supervised machine learning (ML) classification models. In this method, ground-truth data of inertial measurements coupled with verified behaviours are collected, typically through observations of the animals fitted with data loggers; these data are used for training the ML model, which is then utilized to classify unobserved inertial data to behavioural modes. This procedure has become a standard tool in bio-logging studies [5, 6], yet it contains several design choices that can potentially impact the accuracy of the model, and hence the validity of results that are based on its output [7].

An important choice is whether to include or exclude mixed segments that contain more than a single behaviour in the ML model training data. Mixed behaviour segments in ground-truth data occur when the focal animal switches behaviour during the observation, and thus the recorded inertial data reflects more than a single behaviour. One approach for dealing with such behaviour switches is to use a change point detection algorithm to detect a change in behaviour based on the statistics of the signal, allowing to split the data into single behaviour segments (of varying time duration). This approach has been demonstrated by Bom et al. [8] but the relatively sophisticated and heavy computation involved render it outside standard use in ecology. Instead, the common practice is to work with fixed time segments and ignore the mixed segments when training the ML classification model (see literature summary in the Appendix, Table S1). However, it is not clear if disregarding mixed segments is the optimal choice, and this question has never been examined systematically despite the potential impact on model accuracy.

There are potential advantages and disadvantages for excluding or including mixed behaviour segments during model training. Excluding them leaves just pure segments and eliminate ‘noisy’ ones that combine data from more than a single behaviour. The mixed segments are likely to be less distinctive in terms of data features, and hence using them for model training might impede model ability to discriminate between the different classes and thus reduce accuracy. However, including the mixed segments may provide more realistic training data, allowing the model to learn segments of switches between behaviours and then perform better when required to classify them in the actual, unobserved data that needs to be classified.

Here, we aim to answer the question whether the inclusion of mixed behaviour segments in training data improves ML model accuracy, and whether this is dependent on proportion of mixed segments in the data, degree of mixture within the segments (see methods), training data sample size, and the segment duration. A secondary question is whether training the model with both mixed and pure segments hamper its performance in classifying pure segments compared to training without mixed segments. This question is targeted to a specific scenario in which training data contains a higher degree of mixed segments than the unobserved data to be classified. This specific, yet not uncommon scenario may occur when training data is collected in captivity and the model is used for classifying behaviours in the wild (e.g., [7, 9, 10]). This is because behaviours in captivity may be much more interrupted due to space limitation or other disturbances, yielding a higher degree of mixed behaviour segments; for an example, consider the continuity of a mammal running behaviour in an enclosure versus in the wild.

To examine whether inclusion of mixed behaviours in model training is beneficial for accuracy we used a data simulation approach based on ground-truth data of body acceleration matched with known (observed) behaviours from four wild species: Damaraland mole-rats *Fukomys damarensis*, meerkats *Suricata suricatta*, olive baboons *Papio anubis*, and polar bears *Ursus maritimus*. In our simulations, ML models (random forest) were trained while including or excluding mixed segments and their performance were then tested on test data either including or not-including mixed segments. These simulations were performed separately per species’ dataset and under four different data scenarios that manipulated the: (1) proportion mixed segments in the data, (2) degree of behavioural mixture in mixed segments—proportion duration of main behaviour in the segment (see methods), (3) duration of segments (0.5–3 s), and (4) sample size. This allows us to investigate if, and under which data conditions, mixed segments inclusion in training enhanced model accuracy, and evaluate the robustness of the findings.

## Methods

We obtained four datasets of body-acceleration data matched with known (observed) behaviours that were collected in four study species: Damaraland mole-rats, meerkats, olive baboons, and polar bears (details below). These datasets were used (separately) as ground-truth data to examine the accuracy of ML models in classifying body-acceleration data to behaviours under different data scenarios (specified below), designed for examining the research questions.

### Classification of body-acceleration data to behaviours

To classify body acceleration data to behaviour, the continuous acceleration data were first sampled into fixed, 2-s segments (unless specified otherwise). Using short, fixed time segments (of usually 1–3 s) is the typical method when classifying body acceleration data to behaviours. The data segments included either a single behavior—pure segments, or more than one behavior—mixed segments, and in this case the labelled behaviour was chosen to be the one of the highest duration. For each segment of acceleration data, 55 statistics were computed (e.g., Mean, Median, Standard Deviation, see full list in [7]) and used as input for the ML models (see [5] for detailed manual of this process). The data were randomly divided into two even partitions: training and test data (We note that since we are able to generate as many random repetitions as we want, this split provides a better estimation of performance than a single k-fold cross-validation). Lastly, the random forest models (random forest algorithm with 250 trees), implemented with scikit-learn package [11] in Python, were trained using the train partition only, and their accuracy results were tested and reported based on the test partition only. Accuracy is measured as the fraction of the test set predicted correctly by the model.

### Data simulations

The main purpose of the data simulations was to test whether training ML models with mixed segments will enhance model accuracy when mixed segments are present in the test data. In the first set of simulations, we sampled 200 pure, 2-s segments from each of the behaviours per each species’ dataset, and gradually added an increasing proportion of mixed segments, ranging from 5 to 30% of the data, in 2.5% increments. We used only mixed segments for which the top behaviour comprises at least 70% of the segment length. At each simulation, we partitioned the data to train and test (see above), trained a first classifier (ML model) just on pure segments and a second classifier on the entire training data (pure + mixed), and both classifiers were then tested either on only the pure segments in the test data, or on the entire test data (pure + mixed). In total, for each simulation, i.e., per each value of percent mixed segments, we obtained 4 accuracy scores representing training with/without mixed segments and test with/without mixed segments. This series of simulations was repeated 20 times to robustly collect statistics of model accuracy across random samples. In all simulations, here and elsewhere, the sampling of the data segments is conducted without replacements.

The rest of the simulation sets are designed to test the robustness of the findings under variation in other key parameters including: the extent of mixing in mixed segments, segment length and sample size. Thus, we run three additional sets of data simulations, in which the procedure is the same as described above (including classifier training and testing, and repetitions), but proportion mixed segment was set to a fixed value of 20% and instead another parameter varies throughout the simulation series.

In the second set of simulations, the inclusion criterion for the mixed segments varies such that the top behaviour must include from 40 to 80% of the segment duration, in increments of 5%. We note that the two lower values allow the top behaviour to be less than half the time, which is possible if there are at least three distinct behaviours during the segment. In the third set of simulations, segment lengths were tested in the range of 0.5 and 3.0 s with 0.5 s increments. In the fourth set of simulations, we test the effect of sample size and change the number of pure observations sampled per behaviour in the range of 50 to 300, in increments of 50 (20% mixed segments are added on top in the case of mixed training simulations). Except from in this fourth simulation series, a sample size of 200 data segments per behavior is chosen as default because this is the maximal amount that we have enough data for across all simulation conditions, particularly, when increasing the segment length or the minimum duration of the top behavior that reduces available sample size from 300 to 200. The ML procedure, including classifier training and testing, and repetitions were performed identically to the first simulation set.

### Datasets of body acceleration matched with behaviours

In this study we analyzed the following datasets of animal body acceleration matched with known (observed) behaviours obtained in four different species.

#### Damaraland mole-rats

We obtained a dataset from 16 Damaraland mole-rats (DMRs) that were collared with acceleration loggers (Technosmart LTD, Italy) for 1–3 weeks, and videotaped during this period to match the acceleration records with known behaviours (see [7] for more details on the collaring procedure). Acceleration was recorded by the loggers continuously at 25 Hz in each of three perpendicular axes. The collaring and videotaping took place in a laboratory facility in the southern Kalahari (Kuruman River Reserve, South-Africa), built of transparent tubes, allowing to observe the DMRs behaviours [12, 13]. We recorded 57, 10-min videos of the collared individuals and labelled the behaviours when they were clearly visible using the Boris software [14]. The ACC data were then coupled with labelled behaviours. The synchronisation of the ACC data with the videotaped labelled behaviours was done by first using the timestamps in the videos and data and then, the data and video were visualized in parallel and distinctive behavioural changes such as the onset of movement after seconds of motionless were used to refine the time synchronization. Only the most frequent behaviours were included in the analysis, which were: Dig, Eat, Stand, Forward Locomotion, Sweep, Rest (see [7] for more details). All research activities on the DMRs including the housing and collaring were done with approval of University of Pretoria Animal Ethics Committee (permits EC089-12, SOP-004-13, EC059-18).

#### Meerkats

We obtained a dataset from 10 wild, juvenile meerkats that were collared with acceleration loggers (Technosmart LTD, Italy) for 2 weeks, between 2018 and 2020. These meerkats are part of a long-term study population in the Kuruman River Reserve [15]. Juveniles at this population are regularly trapped at 3-month age for monitoring and blood sampling under anesthesia and during this procedure the study animals were equipped with leather collars with 3-g acceleration logger, which is far below 3% of their body weight (~ 300 g, [16]). Collar removal was done by cutting it off without trapping which is feasible in this study population which highly habituated to human presence [15]. Acceleration was recorded by the loggers continuously at 50Hz in each of three perpendicular axes. Twenty-one, 10-min videos were recorded by following these collared juveniles in the wild. The ACC data were then coupled with the behaviours manually by using the time stamps in the videos and in the data and refining their synchronization manually. This manual synchronization was done by visualizing the videos using VLC media player (VideoLAN, Paris, France, with a specific add-on ‘Time v3.2’ allowing display of seconds and milliseconds) in parallel of visualizing the ACC data using the ‘Daily Diary Multi-Trace’ (DDMT) software (Wildbytes Ltd., Swansea, UK) and relying on distinctive behaviours such as ‘vigilance’ (involving standing up on two legs) to refine the time synchronization matching. Only the most frequent behaviours were included in the analysis, which were: Scrabbling, Foraging, Vigilance, Digging, Walking, Running. All study procedures were approved by the Animal Ethics Committee of the University of Pretoria, South Africa (no. EC047-16) and by the Northern Cape Department of Environment and Nature Conservation, South Africa (FAUNA 1020/2016).

#### Olive baboons

We used a dataset from 6 olive baboons that were collared with data loggers (e-Obs Digital Telemetry, Gruenwald, Germany) in August 2019 for a period of one month and videotaped during this period to match the acceleration records with known behaviours. Continuous triaxial accelerations were recorded at 12 Hz/axis from 06:00 to 18:00, and likewise 35 h of videos to match acceleration measurements with verified behavioural modes resulting in 2.5 h of matched and annotated data. Only the most frequent behaviours were included in this analysis, which were: Forage, Sit, Stand, Run, Walk and Vigilant. See more details on data acquisition and permits in [17].

#### Polar bears

We used a dataset from 5 polar bears that were collared by Pagano et al. [18] with data loggers (TDR10- X-340D, Wildlife Computers) and videotaped during this period to match the acceleration records with known behaviours (see [18] for more details). The data were downloaded from open-access dataset published by [19]. Acceleration was recorded by the loggers continuously at 16Hz in each of three perpendicular axes and likewise over 1000 h of videos from the same 5 collared individuals were obtained in order to match acceleration measurements with verified behavioural modes. Only the most frequent behaviours were included in the analysis, which were: Laying, Walking, Eating, Swimming, Digging, Grooming. See more details on data acquisition and permits in [18].

## Results

**Fig. 1:**
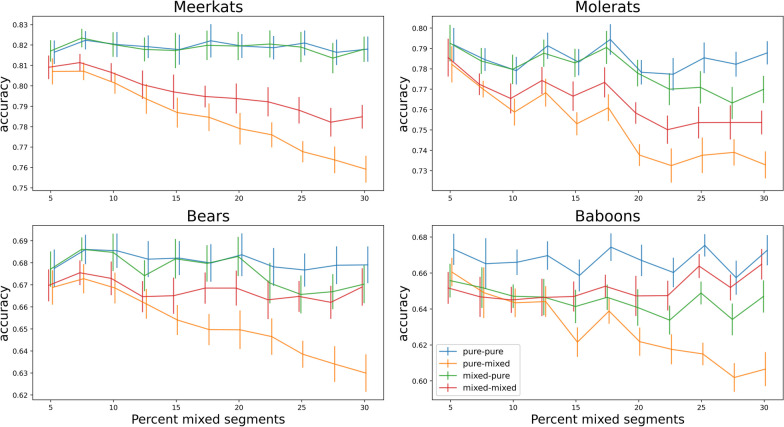
Effect of the percent of mixed segments on classifier accuracy. Bars indicate 95% confidence intervals and solid lines the average. Simulation conditions—pure-pure (blue): train and test with pure, single-behaviour segments only; pure-mixed (orange): train with pure only and test with mixed and pure; mixed-pure (green): train with mixed and pure and test with pure only; mixed-mixed (red): train and test with mixed and pure segments. The relevant comparisons are between conditions (lines) of same test type: mixed-mixed (red) versus pure-mixed (orange), and mixed-pure (green) versus pure-pure (blue)

The effect of including mixed behaviour segments during model training depends on the composition of the test data (Fig. 1). When test data consists of both mixed and pure segments, training the model with mixed segments improves its accuracy relative to training without them; yet, this improvement is dependent on the proportion of mixed segments: it emerges above ~ 10% and gradually increases thereafter, a trend which is consistent across the studied species (Fig. 1). We also found this to be true regardless of segment length (See Supplementary Figures S1–S2). When testing with pure segments only, the effect of adding in mixed segments during training on model accuracy is usually negative (in three of the four study species) but small even at max level of proportion mixed segments (Fig. 1). I.e., including mixed segments in training slightly reduces model accuracy compared to training without them when test data includes only pure segments.

**Fig. 2:**
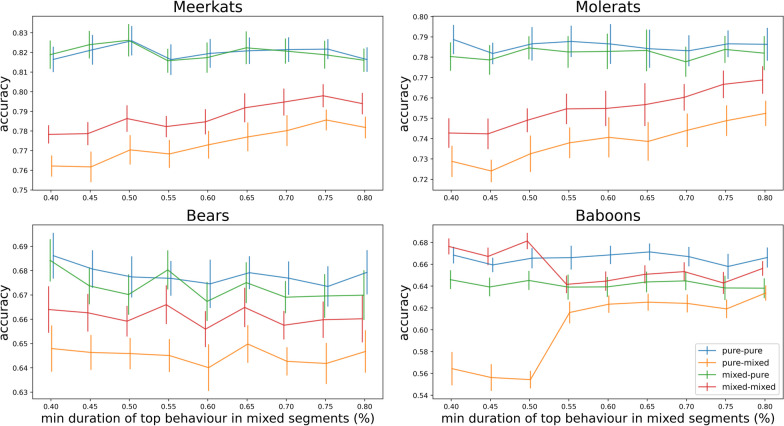
Effect of the extent of mixing (minimum duration of the top behaviour in each mixed segment) on classifier accuracy (20% of segments are mixed). Bars indicate 95% confidence intervals and solid lines the average. Simulation conditions—pure-pure: train and test with pure, single-behaviour segments only; pure-mixed: train with pure only and test with mixed and pure; mixed-pure: train with mixed and pure and test with pure only; mixed-mixed: train and test with mixed and pure segments

When the degree of mixture inside the mixed segments increases, the accuracy of classifying them decreases, however across all levels of mixture, when mixed segments are present in test data the trend of accuracy improvement from having them also in train data remains (Fig. 2).

**Fig. 3:**
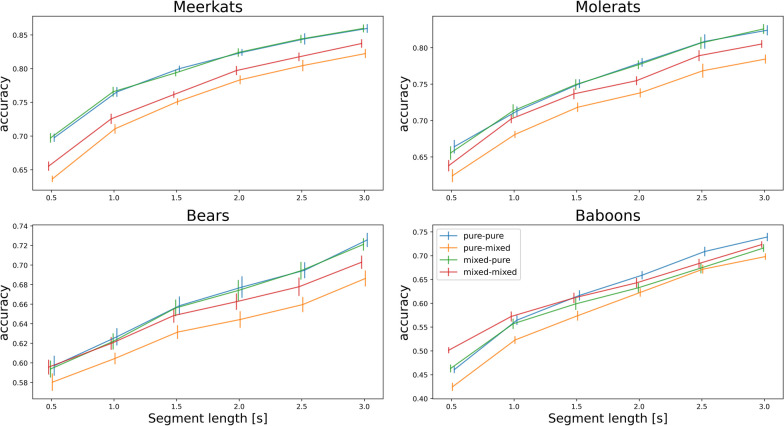
Effect of segment length on classifier accuracy (20% of segments are mixed). Bars indicate 95% confidence intervals and solid lines the average. Simulation conditions—pure-pure: train and test with pure, single-behaviour segments only; pure-mixed: train with pure only and test with mixed and pure; mixed-pure: train with mixed and pure and test with pure only; mixed-mixed: train and test with mixed and pure segments

**Fig. 4:**
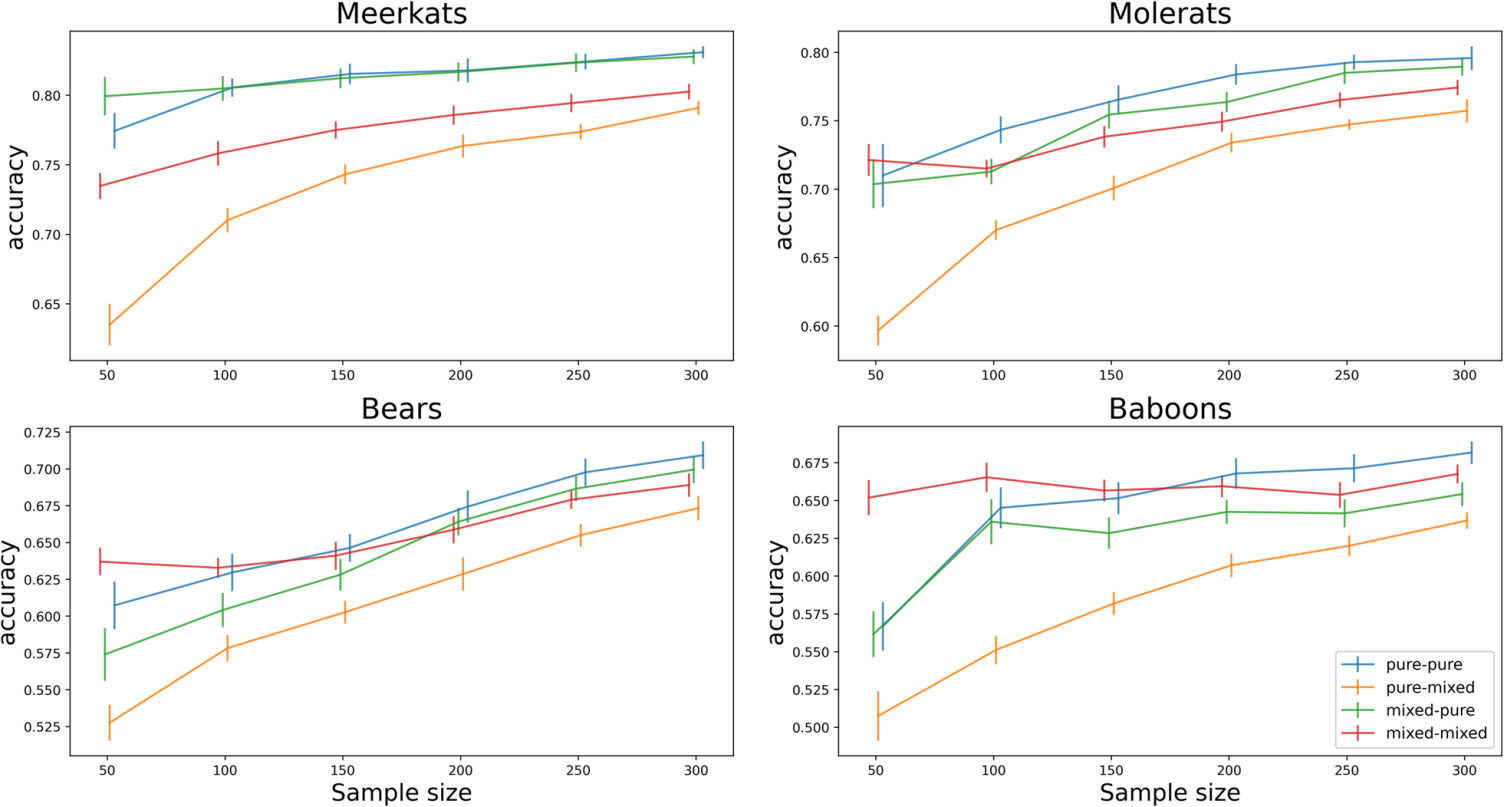
Effect of sample size per behaviour on classifier accuracy (20% of segments are mixed). Bars indicate 95% confidence intervals and solid lines the average. Simulation conditions—pure-pure: train and test with pure, single-behaviour segments only; pure-mixed: train with pure only and test with mixed and pure; mixed-pure: train with mixed and pure and test with pure only; mixed-mixed: train and test with mixed and pure segments

We also find an overall gradual increase in classification accuracy with an increase in segment length (Fig. 3) and sample size (Fig. 4). Also here, throughout the tested parameter ranges we find consistency in the trends reported above of (a) improved classification accuracy in test phase with mixed segments when the model training data included them and (b) slightly reduced accuracy in test phase with only pure segments when the model was trained also with mixed ones.

## Discussion

When behavioural modes are classified from inertial measurement data using a supervised machine learning model (classifier), there are several important design choices researchers make, which have the potential to affect the accuracy of the classification. Using data simulations, we investigate how the inclusion of mixed behaviour segments during the training phase affects the accuracy of the behavioural inference during model predictions. We found that when mixed segments constitute more than 15% of the data, their inclusion in model training improved classification accuracy (Fig. 1), a finding that was consistent across the different species’ datasets and data scenarios that were examined. For this reason, we generally recommend including mixed segment in ML model training particularly when their proportion in the data is substantial (~ > 15%). When mixed segment proportion is roughly below 10%, our findings show that including them in model training phase has no effect on model accuracy. However, our findings also show that inclusion of mixed segments in training data can moderately reduce accuracy in classifying pure (single behaviour) segments. Thus, if there is a basis to suspect that the proportion of mixed segments is higher in the training dataset than in the classified data, their inclusion needs to be considered cautiously.

Mixed behaviour segments are typically ignored in animal behaviour studies that translate sensor data to behavioural modes. Mixed segments are usually not mentioned in such studies (Table S1) even though it is unlikely that they do not occur at all as animals regularly transit between behaviours, generating such segments. In a few studies it is accurately reported that only pure segments are extracted [20, 21], or that mixed segments are excluded [22, 23]. Our results suggest that this common approach might be suboptimal, particularly when mixed segments proportion is considerable. Furthermore, accuracy estimates are possibly inflated when they are reported based on the pure-pure calculation, when in fact the model is later used on mixed segments. Thus, we generally suggest to add mixed segments to model training and testing phases, examine if this improves accuracy (as we find here), and based on that decide how to treat the mixed segments.

The reduction in accuracy in classification of pure segments during testing when mixed segments were used for training can be explained intuitively; when mixed segments are added to model training, the model learns less discriminative patterns of the behavioural classes with negative implication for classification accuracy when applied to pure segments. Thus, when the models were tested only on pure segments, inclusion of mixed segments in training resulted in moderate reductions in accuracy in three out of four examined species’ datasets (Fig. 1). This finding suggests that if training data exceeds test data in mixed segments proportion, it is better to leave out the excess mixed segments from model training. This suggestion is particularly relevant for studies in which training data is collected on captive animals and used to classify data from wild individuals—a scenario which is common when it is difficult to observe the animal in the wild (e.g., [7, 9, 10]). In such scenarios, if it is reasonable to assume and/or there are data indications that the observed behaviours in the training data (captivity) are more interrupted than in the data to be classified (wild), one should consider cautiously, and possibly avoid, the inclusion of mixed segments in model training.

The chosen segment duration that is used for the ML classification encapsulates a trade-off between a time window that will comprise only a single behaviour yet will be long enough contain sufficient data to discriminate between behaviours [4, 24], and our suggestion to include mixed segments in the classification model training can affect this decision. Short data segments, e.g. of one second [9, 25, 26], would favorably provide relatively more pure segments and less segments with behaviour transition. On the other hand, too short time window may not include sufficient data to discriminate accurately between similar behaviours. Our findings show that classification accuracy when using short time segments of only pure behaviours is comparable to using longer time window and accounting for mixed segments by their inclusion in model training (Fig. 3; lines: pure-pure vs. mixed-mixed). Therefore, in the trade-off between short pure segments and longer ones having more data per segment, our analysis offers to consider an in-between option of using longer segments that contains a larger proportion of mixed segments, if the model can account for them accurately through training on an adequate training set (with mixed segments).

When mixed behaviour segments are used in ML model training, it needs to be decided which behaviour the mixed segment will be assigned to, i.e., which behavioural label will be used in the training phase. Here, we defined this behaviour to be the one of the longest duration. Alternative decision rules can be used such as to decide based on the behaviour of higher ecological importance to the research question (as in: [27, 28]). For example, a study that is focused on pecking behaviour may assign mixed pecking-standing segments as ‘pecking’ even if they mostly comprise of standing.

It is commonly understood to be advantageous to use as much training data as possible when training models for supervised learning of behavioural modes from accelerometer data. Correspondingly, our results (Fig. 4) show an overall trend of increase in accuracy in all species as the sample size is increased. Also, the main effect of benefiting from adding mixed segments to training when later needing to classify such is seen consistently throughout the entire range of sample size studied here. In the Baboons dataset we see that when the sample size is small, the effect of adding in the mixed segments is stronger, and generally attenuates gradually as the sample size is increased. One reason for this could be that for small sample sizes the increase in training data represented by adding in an additional 20% of mixed segments is more crucial for improving accuracy than for sample size that is already larger.

### Concluding recommendations

First, we recommend reporting whether there are mixed behaviour segments in ground-truth sensor data matched with behaviours that is collected for ML classification, and their proportion. Based on the trends in our simulations on data from four mammal species, we conclude that if mixed segment proportion is below 10% of the data, it is probably not worthwhile in terms of accuracy to include them in ML model training (yet this could still be easily examined per study). If mixed segment proportion is above 15%, we recommend including, or consider their inclusion, in model training and testing. Nevertheless, if there are reasons to suspect that mixed segment proportion is higher in the ground-truth data than in the actual data to be classified (e.g., captivity versus wild case—see above), their inclusion needs to be considered cautiously and excess inclusion of mixed segments should probably be avoided. 

### Supplementary Information

Supplementary Material

## Data Availability

Data will be provided upon request and made public with the publication.
