# Supplementary material for: How to treat mixed behavior segments in supervised machine learning of behavioural modes from inertial measurement data
Source: Mov Ecol. 2024 Jun 10;12:44. doi: 10.1186/s40462-024-00485-7 (PMC11165886; doi:10.1186/s40462-024-00485-7)
Supplement: Supplementary file 1 — Supplementary Material [file 40462_2024_485_MOESM1_ESM.docx]

# Appendix

**Table S1: Literature review**

The following table summarizes a limited literature review examining how mixed segments (of more than a single behavior) were treated in recent papers utilizing supervised machine learning to classify body-acceleration to behavioral modes. More specifically, we examined whether mixed segments were used in the machine learning training phase. The *treatment* column (first column) in the table reflects that. The list of papers was obtained through the ‘Web Of Science’ website (<https://www.webofscience.com/wos>) by conducting a search on the ‘core collection’ using the query: ‘(body acceleration) AND (behavior) AND (animal)’. The search was conducted on 20-Oct-2023, and the resulting list was then refined for the categories: ‘ecology’ and ‘zoology’, yielding a list of 180 papers. We scanned this list to extract only the relevant papers, that conducted classification of body-acceleration to behavioral modes via supervised machine learning models (n=16) and added another four relevant papers based on an anonymous reviewer's comment or our previous knowledge(these are: ([Fehlmann et al. 2017](#_ENREF_6), [Chakravarty et al. 2019](#_ENREF_2), [Arkumarev et al. 2020](#_ENREF_1), [Giese et al. 2021](#_ENREF_8))).

| **Mixed segments treatment** | **Count** | **Papers** |
| --- | --- | --- |
| Not mentioned – probably ignored^1^ | 15 | ([Génin et al. 2015](#_ENREF_7), [Rotics et al. 2016](#_ENREF_13), [Fehlmann et al. 2017](#_ENREF_6), [Rotics et al. 2018](#_ENREF_14), [Arkumarev et al. 2020](#_ENREF_1), [Clarke et al. 2021](#_ENREF_3), [Dickinson et al. 2021a](#_ENREF_4), [Dickinson et al. 2021b](#_ENREF_5), [Giese et al. 2021](#_ENREF_8), [Rotics et al. 2021](#_ENREF_15), [VonBank et al. 2021](#_ENREF_19), [Menzies et al. 2022](#_ENREF_10), [Resheff et al. 2022](#_ENREF_12), [Hanscom et al. 2023](#_ENREF_9), [Soriano-Redondo et al. 2023](#_ENREF_16)) |
| Stating that pure segments were used | 2 | ([Weegman et al. 2017](#_ENREF_20), [Thiebault et al. 2021](#_ENREF_18)) |
| Excluding mixed segments explicitly | 2 | ([Chakravarty et al. 2019](#_ENREF_2), [Pagano et al. 2020](#_ENREF_11)) |
| There were no mixed segments^2^ | 1 | ([Studd et al. 2021](#_ENREF_17)) |
| Including mixed segments | 0 |  |

^1^ The most common case is not mentioning the mixed segments, and since it is unlikely that they do not occur at all, we consider this as ignoring them in the analysis. ^2^ Stating that there were no mixed segments owing to using very short time window in the analysis.

**S2: The interaction between interaction percent mixed segments and segment length**

In order to test the interaction between the percent mixed segments and segment length, we repeated the first set of simulations (see Methods) for a range of values of segment length, using the Meerkats dataset. Each combination was repeated 20 times. Results are presented as a heatmap of the effect of using mixed segments during training when they appear also during test (the mixed-mixed versus pure-mixed conditions). In addition, we show a heatmap of the p-values associated with these effects, using a one-tailed t-test for each combination of percent mixed and segment length, over the 20 simulation repetitions.

Results (Figure S2-1) show substantial effects wherever percent mixed is 15% or above. This is also reflected in the statistical significance of the effects (Figure S2-2). These results are generally consistent with the main findings presented for the case of 2-second segments.


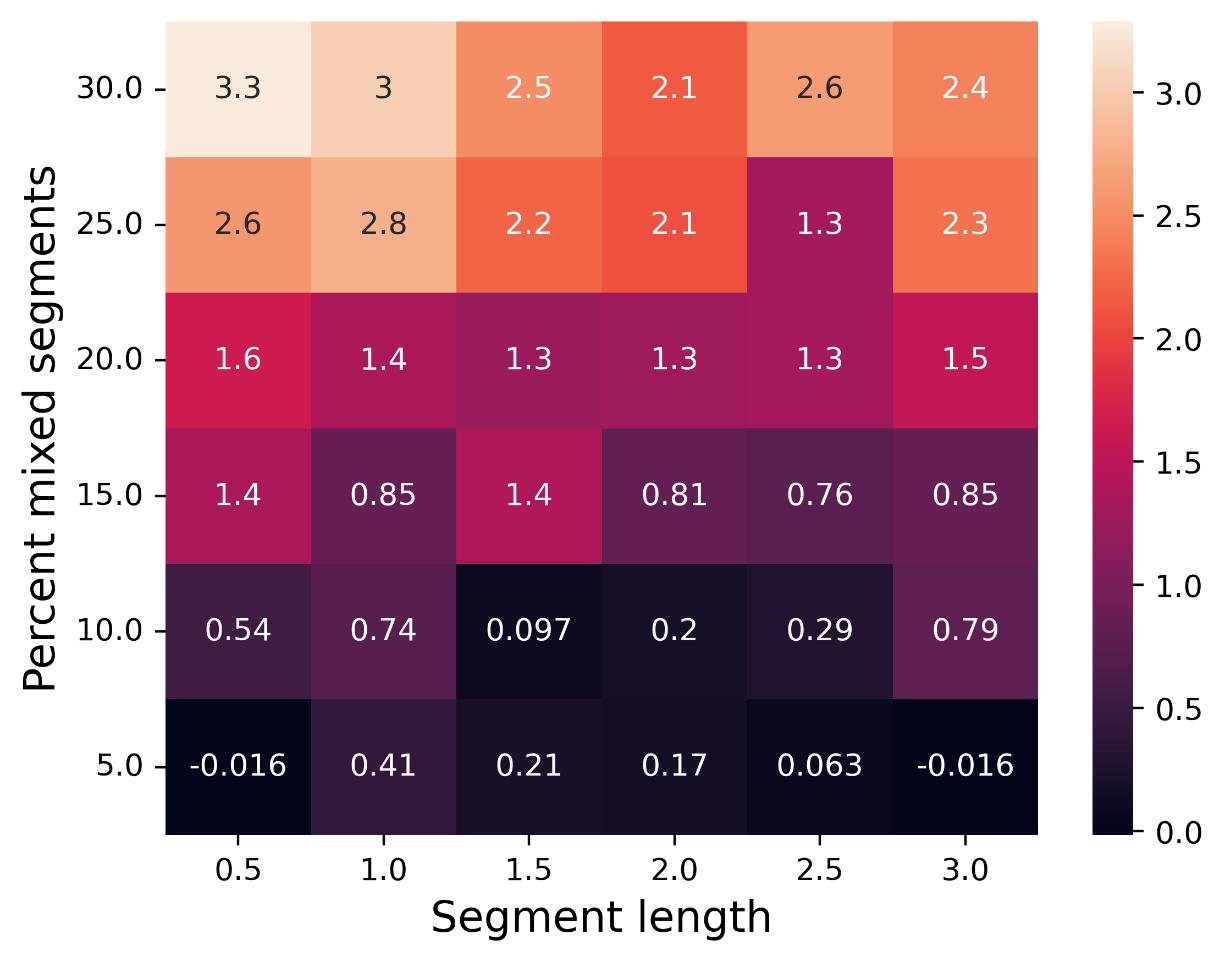


Supplementary Figure 1: Effect of the percent of mixed segments and segment length on classifier accuracy. Values indicate the average effect over the 20 repetitions.


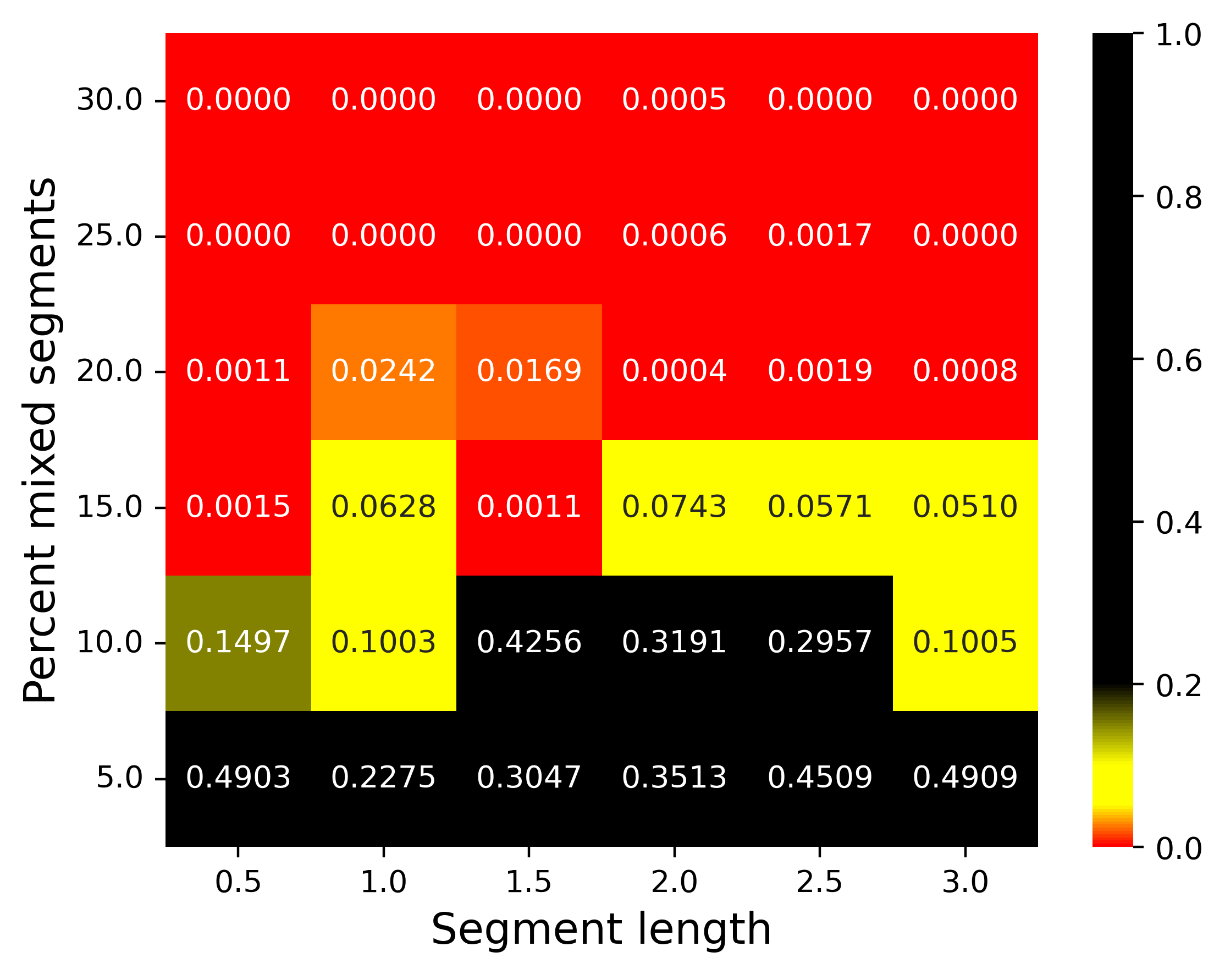


Supplementary Figure 2: Statistical significance of the effect of the percent of mixed segments and segment length on classifier accuracy. Values indicate the p-value for the one-sided t-test with an alternative hypothesis of mixed training being beneficial.

**Table S1references**

Arkumarev, V., D. Dobrev, A. Stamenov, N. Terziev, A. Delchev, and S. Stoychev. 2020. Using GPS and accelerometry data to study the diet of a top avian scavenger. Bird Study **67**:300-310.

Chakravarty, P., G. Cozzi, A. Ozgul, and K. Aminian. 2019. A novel biomechanical approach for animal behaviour recognition using accelerometers. Methods in Ecology and Evolution **10**:802-814.

Clarke, T. M., S. K. Whitmarsh, J. L. Hounslow, A. C. Gleiss, N. L. Payne, and C. Huveneers. 2021. Using tri-axial accelerometer loggers to identify spawning behaviours of large pelagic fish. Movement Ecology **9**.

Dickinson, E. R., P. A. Stephens, N. J. Marks, R. P. Wilson, and D. M. Scantlebury. 2021a. Behaviour, temperature and terrain slope impact estimates of energy expenditure using oxygen and dynamic body acceleration. Animal Biotelemetry **9**.

Dickinson, E. R., J. P. Twining, R. Wilson, P. A. Stephens, J. Westander, N. Marks, and D. M. Scantlebury. 2021b. Limitations of using surrogates for behaviour classification of accelerometer data: refining methods using random forest models in Caprids. Movement Ecology **9**.

Fehlmann, G., M. J. O’Riain, P. W. Hopkins, J. O’Sullivan, M. D. Holton, E. L. C. Shepard, and A. J. King. 2017. Identification of behaviours from accelerometer data in a wild social primate. Animal Biotelemetry **5**:6.

Génin, A., G. Richard, J. Jouma'a, B. Picard, N. El Ksabi, J. V. Garcia, and C. Guinet. 2015. Characterization of postdive recovery using sound recordings and its relationship to dive duration, exertion, and foraging effort of southern elephant seals (<i>Mirounga leonina</i>). Marine Mammal Science **31**:1452-1470.

Giese, L., J. Melzheimer, D. Bockmühl, B. Wasiolka, W. Rast, A. Berger, and B. Wachter. 2021. Using Machine Learning for Remote Behaviour Classification-Verifying Acceleration Data to Infer Feeding Events in Free-Ranging Cheetahs. Sensors **21**.

Hanscom, R. J., D. L. DeSantis, J. L. Hill, T. Marbach, J. Sukumaran, A. F. Tipton, M. L. Thompson, T. E. Higham, and R. W. Clark. 2023. How to study a predator that only eats a few meals a year: high-frequency accelerometry to quantify feeding behaviours of rattlesnakes (<i>Crotalus</i> spp.). Animal Biotelemetry **11**.

Menzies, A. K., E. K. Studd, J. L. Seguin, R. E. Derbyshire, D. L. Murray, S. Boutin, and M. M. Humphries. 2022. Activity, heart rate, and energy expenditure of a cold-climate mesocarnivore, the Canada lynx (Lynx canadensis). Canadian Journal of Zoology **100**:261-272.

Pagano, A. M., T. C. Atwood, G. M. Durner, and T. M. Williams. 2020. The seasonal energetic landscape of an apex marine carnivore, the polar bear. Ecology **101**.

Resheff, Y. S., H. M. Bensch, M. Zöttl, and S. Rotics. 2022. Correcting a bias in the computation of behavioural time budgets that are based on supervised learning. Methods in Ecology and Evolution **13**:1488-1496.

Rotics, S., M. Kaatz, Y. S. Resheff, S. F. Turjeman, D. Zurell, N. Sapir, U. Eggers, A. Flack, W. Fiedler, F. Jeltsch, M. Wikelski, and R. Nathan. 2016. The challenges of the first migration: movement and behaviour of juvenile vs. adult white storks with insights regarding juvenile mortality. Journal of Animal Ecology **85**:938-947.

Rotics, S., M. Kaatz, S. Turjeman, D. Zurell, M. Wikelski, N. Sapir, U. Eggers, W. Fiedler, F. Jeltsch, and R. Nathan. 2018. Early arrival at breeding grounds: Causes, costs and a trade-off with overwintering latitude. Journal of Animal Ecology **87**:1627-1638.

Rotics, S., S. Turjeman, M. Kaatz, D. Zurell, M. Wikelski, N. Sapir, W. Fiedler, U. Eggers, Y. S. Resheff, F. Jeltsch, and R. Nathan. 2021. Early-life behaviour predicts first-year survival in a long-distance avian migrant. Proceedings of the Royal Society B-Biological Sciences **288**.

Soriano-Redondo, A., A. M. A. Franco, M. Acácio, A. Payo-Payo, B. H. Martins, F. Moreira, and I. Catry. 2023. Fitness, behavioral, and energetic trade-offs of different migratory strategies in a partially migratory species. Ecology.

Studd, E. K., R. E. Derbyshire, A. K. Menzies, J. F. Simms, M. M. Humphries, D. L. Murray, and S. Boutin. 2021. The Purr-fect Catch: Using accelerometers and audio recorders to document kill rates and hunting behaviour of a small prey specialist. Methods in Ecology and Evolution **12**:1277-1287.

Thiebault, A., C. Huetz, P. Pistorius, T. Aubin, and I. Charrier. 2021. Animal-borne acoustic data alone can provide high accuracy classification of activity budgets. Animal Biotelemetry **9**.

VonBank, J. A., M. D. Weegman, P. T. Link, S. A. Cunningham, K. J. Kraai, D. P. Collins, and B. M. Ballard. 2021. Winter fidelity, movements, and energy expenditure of Midcontinent Greater White-fronted Geese. Movement Ecology **9**.

Weegman, M. D., S. Bearhop, G. M. Hilton, A. J. Walsh, L. Griffin, Y. S. Resheff, R. Nathan, and A. D. Fox. 2017. Using accelerometry to compare costs of extended migration in an arctic herbivore. Current Zoology **63**:667-674.
